# Assessment and Comparison of Natural Dyes Like Lawsonia inermis, Zingiber officinale, Curcuma longa, Beta vulgaris, Kumkum, and Hibiscus rosa-sinensis With Eosin as a Cytoplasmic Stain in Oral Histopathologies

**DOI:** 10.7759/cureus.79200

**Published:** 2025-02-18

**Authors:** G Deepthi, D Shyam Prasad Reddy, S Keerthi Sai, Mary Oshin

**Affiliations:** 1 Oral and Maxillofacial Pathology, Kamineni Institute of Dental Sciences, Narketpally, IND; 2 Oral and Maxillofacial Pathology, Indira Gandhi Institute of Dental Sciences, Kothamangalam, IND

**Keywords:** curcuma longa, eosin, hibiscus, lawsonia inermis, natural stains

## Abstract

Background

Hematoxylin and eosin (H&E) staining is a commonly used technique in histology and histopathology. However, the synthetic dye eosin poses risks to both human and animal health. The growing ecological concern has led to efforts to find natural, less toxic, biodegradable, and cost-effective alternatives to eosin.

Aim

This study aimed to compare and evaluate the staining characteristics of natural dyes as cytoplasmic stain to eosin as a standard in oral histopathologies.

Materials and methods

A total of 140 histological sections from four tissue types (normal mucosa, oral squamous cell carcinoma, ameloblastoma, and odontogenic cysts) were stained with natural dyes. The groups were divided based on the type of stain: Group A (*Lawsonia inermis*), Group B (*Zingiber officinale*), Group C (*Curcuma longa*), Group D (kumkum), Group E (*Hibiscus rosa-sinensis*), Group F (*Beta vulgaris*), and Group G (eosin as control). The staining intensity and nuclear and cytoplasmic detail were evaluated and compared using one-way ANOVA. A significance level of 0.01 was set for statistical analysis.

Results

One-way ANOVA revealed a significant difference in staining intensity across the seven groups (p=0.001). Eosin showed the highest mean staining intensity (15.85), followed by *Lawsonia inermis* (15.05), while *Hibiscus rosa-sinensis *and *Beta vulgaris* displayed the lowest values (7.9 and 8.4, respectively). Post-hoc analysis identified Group A (mean=15.05) shows no significant difference from the control suggesting that the staining performance of Group A is comparable to the control at (p<0.01).

Conclusion

The utilization of *Lawsonia inermis* as a natural dye in histopathological staining procedures has demonstrated promising results compared to other natural stains. Its ability to provide excellent contrast, detailed cellular and nuclear staining, and effective staining of acellular structures, comparable to eosin, underscores its potential as a viable alternative in diagnostic pathology.

## Introduction

Dyes are compounds that lend color to other substances. The introduction of dyes and stains has significantly enhanced the precision of cellular tissue differentiation. This is because stains improve optical contrast among cellular components by either modifying contrast or adding color [1]. Dyes can be classified as either natural or synthetic based on their origin.

Utilizing herbs to create dyes is a practice that dates back through human history. Various flowers, roots, leaves, and barks of herbal species have commonly been employed for dye production, often through methods such as boiling, grinding into powder, and blending with other substances [2]. Synthetic dyes, on the other hand, are typically prepared through chemical processes involving the synthesis of organic compounds. While efficient, synthetic dyes pose risks to human and animal health like allergic reactions, toxicity, and potential carcinogenic effects, prompting increased awareness of these hazards and, in some cases, the withdrawal of certain dyes from use. Prolonged exposure to these chemicals can adversely impact the health of laboratory technicians, pathologists, and others working directly in laboratory settings [1,3].

Hematoxylin (H) and eosin (E) dyes, among the vast array of dyes employed in histopathology, remain the gold standard for routine staining. Hematoxylin, sourced from the Mexican tree *Haematoxylum campechianum*, is a natural dye, whereas eosin is synthetic. Although synthetic dyes are generally efficient, their associated health risks have led to the withdrawal of several dyes from use [4].

Despite certain limitations like limited stability and reproducibility, natural stains can be considered alternatives to conventional stains. However, research exploring the use of natural dyes in human tissues remains limited, with minimal data available in the literature. The present study focuses on the utilization of natural dyes in histopathology as opposed to synthetic dyes, considering the drawbacks of the latter. Additionally, this study aims to identify the most effective natural dye and evaluate the diagnostic efficacy of using these natural dyes for staining oral histopathological sections.

Natural dyes such as *Zingiber officinale* (ginger), *Lawsonia inermis* (henna), *Curcuma longa* (turmeric), *Beta vulgaris* (beetroot), kumkum, and *Hibiscus rosa-sinensis* (hibiscus) were compared with eosin as cytoplasmic stains. To the best of our knowledge, this is the first study to compare the staining effectiveness of six natural dyes with eosin across various oral histopathological tissue sections.

## Materials and methods

The study was a comparative analysis conducted in the Histopathology Laboratory of the Department of Oral and Maxillofacial Pathology at the Kamineni Institute of Dental Sciences, Narketpally, India. It was carried out from October 2023 to February 2024, following approval from the institute's Institutional Ethics Committee, under IEC number KIDS/IEC/FACULTY/OP/2023/02.

Sample collection

The study comprised 140 histological sections acquired from the departmental archives. These sections were categorized based on staining groups as follows: Group A stained with *Lawsonia inermis* (henna), Group B with *Zingiber officinale* (ginger), Group C with *Curcuma longa* (turmeric), Group D with kumkum, Group E with *Hibiscus rosa-sinensis* (hibiscus), Group F with *Beta vulgaris* (beetroot), and Group G with eosin as a control. Each staining group included sections from the following cases: five normal tissues, five oral squamous cell carcinoma biopsy tissue specimen blocks, five ameloblastoma biopsy tissue specimen blocks, and five odontogenic cysts tissue specimen blocks, each cut into seven sections (n=35 per case type (five tissue sections × seven stains). The counterstaining for all the natural dyes and eosin was performed using hematoxylin. The staining qualities like cytoplasmic staining, intensity, and contrast were assessed and scored. The extraction of the natural dyes and their staining procedures are explained below.

Extraction of natural dyes/stains and staining procedures

The following natural dyes were extracted using standardized protocols, developed based on a review of existing studies.

Zingiber officinale (Ginger)

Extract: Fresh rhizomes of *Zingiber officinale* were procured and thoroughly washed to eliminate dirt and impurities. Subsequently, the rhizome was peeled and diced into small fragments, and 25 g of these fragments were mixed with 100 ml of 90% alcohol. Following a 24-hour period, the mixture was filtered, yielding 80 ml of *Zingiber officinale* extract for staining purposes [3].

Staining procedure: Tissue sections were dewaxed on a slide warmer and were cleared in xylene for 15 minutes, followed by hydration in 80% alcohol and rinsing in water. Subsequently, they were stained with Harris hematoxylin for five minutes and rinsed in running tap water. Differentiation was carried out in 1% acid alcohol for 2-3 seconds, followed by bluing in running tap water for 15 minutes. Counterstaining was then performed with 90% ethanolic extract of *Zingiber officinale* stain for eight minutes. Finally, sections were rinsed in water, cleared in xylene, and mounted in DPX mountant [3].

Lawsonia inermis (Henna)

Extract: There is no standardized method for the extraction of the dye; therefore, a protocol was developed through multiple trials to achieve successful extraction. Briefly, 50 g of dried *Lawsonia inermis *(henna) powder was mixed with 250 ml of 90% ethanol and left undisturbed for 24 hours. The resulting solution was then filtered using Whatman No. 1 filter paper.

Staining procedure: Tissue sections were initially dewaxed and rehydrated through a graded alcohol series. Staining commenced with Harris hematoxylin for five minutes, followed by rinsing under running tap water. Differentiation was performed using 1% acid alcohol for 2-3 seconds, after which sections were blued under running tap water for 15 minutes. Counterstaining was carried out using the 90% ethanolic extract of *Lawsonia inermis *for 10 minutes. Finally, the sections were rinsed with water, cleared in xylene, and mounted with DPX mountant.

Curcuma longa (Turmeric)

Extract: Twenty grams of the commercially available turmeric powder were dissolved in 100 ml of 70% alcohol and allowed to sit for 24 hours. The supernatant was subsequently separated, yielding 70 ml of *Curcuma longa *extract, which was utilized for staining purposes [3].

Staining procedure: The staining procedure, akin to *Zingiber officinale*, was pursued up to bluing. Following this, the sections were counterstained with 90% ethanolic extract of *Curcuma longa *stain for nine minutes. Subsequently, they were rinsed in water, dehydrated in 80% absolute alcohol, cleared in xylene, and mounted [3].

Beta vulgaris (Beetroot)

Extract: Ten grams of commercially available organic beetroot powder were combined with 100 ml of 80% ethanol and allowed to sit for 24 hours. Following this, a double filtration process was employed using Whatman filter paper. Subsequently, the filtrates were centrifuged at 3000 revolutions per minute (rpm) for 15 minutes. The resulting supernatant was transferred into a reagent bottle and appropriately labeled [2].

Staining procedure: The tissue sections underwent dewaxing in xylene and hydration through descending grades of alcohol (95%, 80%, and 70%). Sections were stained with Harris hematoxylin for five minutes and rinsed in running tap water. Differentiation was carried out in 1% acid alcohol for 2-3 seconds, followed by bluing in running tap water for 15 minutes. Subsequently, they were briefly rinsed in vinegar and immersed in beetroot extract for 30 minutes to one hour. Afterward, the sections were washed in vinegar, followed by a final rinse in water, and cleared in xylene, and mounting was done using DPX [2].

Kumkum

Extract: Fifteen grams of commercial kumkum powder were diluted with 100 ml of 70% alcohol and left undisturbed for 48 hours. The supernatant was filtered using Whatman filter paper, and the resulting filtrates were stored in labeled bottles [5].

Staining procedure: The staining procedure, similar to *Zingiber officinale*, was pursued up to bluing. Following this, the sections were counterstained with 90% ethanolic extract of kumkum stain for nine minutes. Subsequently, they were rinsed in water, dehydrated in 80% absolute alcohol, cleared in xylene, and mounted [5].

Hibiscus rosa-sinensis (Hibiscus)

Extract: Three grams of commercially available organic hibiscus powder were combined with 100 ml of 80% ethanol and heated to 70°C in a water bath for three hours. Following this, the extracts were allowed to cool. To purify the extracts, a double filtration process was employed using Whatman filter paper. Subsequently, the filtrates were centrifuged at 3000 rpm for 15 minutes. The resulting supernatant was transferred into a reagent bottle and appropriately labeled [2].

Staining procedure: The sections were dewaxed and were subjected to various alcohol gradients. Hematoxylin was used for nuclear staining, followed by differentiation with 1% acid alcohol. Subsequently, the sections were stained with alcoholic hibiscus extract for 10 minutes at room temperature, followed by a final rinse in water, cleared in xylene, and mounted using DPX [2].

Scoring criteria

Each section stained with a different natural stain was evaluated and compared to the H&E-stained section based on contrast, as well as the morphological details of the cytoplasm, nucleus, and acellular structures. Each parameter was scored on a scale of 1-4 using the predefined scoring system outlined in Table 1 [5].

**Table 1:** Scoring system for the evaluation of natural stains compared to H&E staining H&E: hematoxylin and eosin

Parameter	Score 1	Score 2	Score 3	Score 4
Contrast (low power, ×100)	Very poor: unsuitable for interpretation	Sub-optimal: just acceptable for interpretation	Optimal contrast at low power	Excellent contrast at low power
Cytoplasmic morphological details	Very poor: unsuitable for interpretation	Sub-optimal: just acceptable for interpretation	Optimal cytoplasmic features	Excellent preservation and sharp cytoplasmic features
Nuclear morphological details	Very poor: nuclear features overtly obscured by counterstain	Sub-optimal: nuclear features moderately obscured by counterstain	Optimal nuclear features: mildly obscured by counterstain	Excellent: nuclear features not obscured by counterstain
Acellular morphological details	Very poor: unsuitable for interpretation	Sub-optimal: just acceptable for interpretation	Optimal morphological features	Excellent preservation and sharp features

The scoring was done for each section based on the above four parameters, and they were tabulated in a Microsoft Excel sheet. The results were then calculated as percentages and analyzed statistically using the ANOVA and post-hoc analysis with IBM SPSS Statistics for Windows, Version 20.0 (Released 2011; IBM Corp., Armonk, New York, United States). A p-value of less than 0.01 was considered statistically significant.

## Results

**Figure 1:**
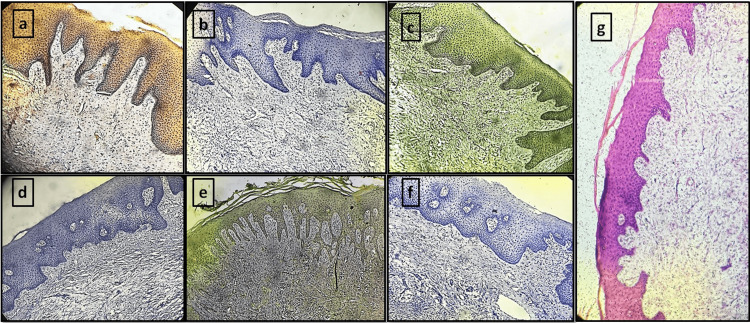
Normal oral mucosa tissue sections stained with different natural dyes and eosin (a) *Lawsonia inermis*. (b) *Zingiber officinale*. (c) *Curcuma longa*. (d) *Hibiscus rosa-sinensis*. (e) Kumkum. (f) *Beta vulgaris*. (g) Eosin (control group)

**Figure 2:**
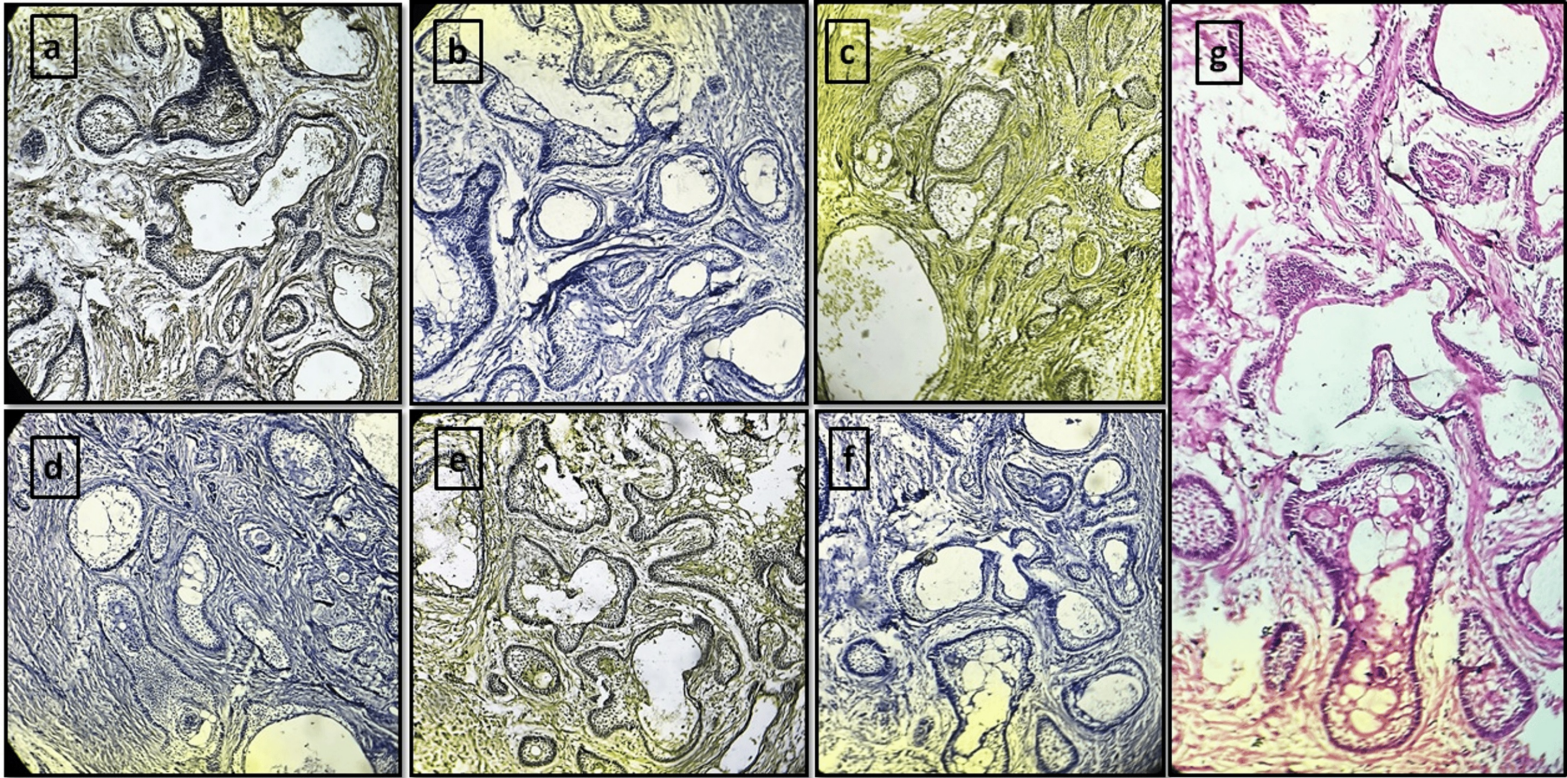
Ameloblastoma tissue sections stained with different natural dyes and eosin (a) *Lawsonia inermis*. (b) *Zingiber officinale. *(c) *Curcuma longa. *(d) *Hibiscus rosa-sinensis. *(e) Kumkum. (f) *Beta vulgaris. *(g) Eosin (control group)

**Figure 3:**
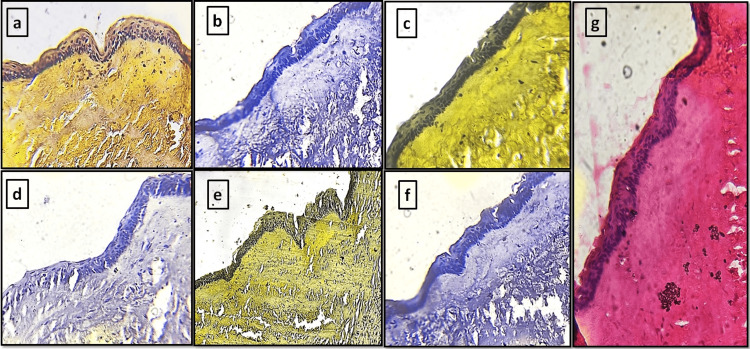
Odontogenic keratocyst tissue sections stained with different natural dyes and eosin (a) *Lawsonia inermis. *(b) *Zingiber officinale. *(c) *Curcuma longa. *(d) *Hibiscus rosa-sinensis. *(e) Kumkum. (f) *Beta vulgaris. *(g) Eosin (control group)

**Figure 4:**
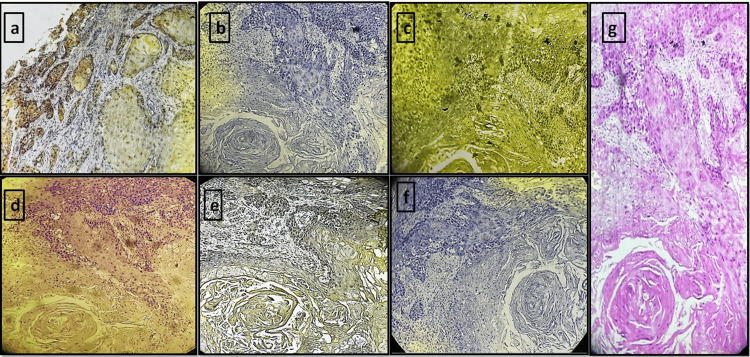
OSCC tissue sections stained with different natural dyes and eosin (a) *Lawsonia inermis. *(b) *Zingiber officinale. *(c) *Curcuma longa. *(d) *Hibiscus rosa-sinensis. *(e) Kumkum. (f) *Beta vulgaris. *(g) Eosin (control group) OSCC: oral squamous cell carcinoma

The present study was conducted on histopathologically diagnosed tissue sections, with each section stained using one of seven different dyes. The staining groups were categorized as follows: Group A, stained with *Lawsonia inermis *(henna); Group B, with *Zingiber officinale *(ginger); Group C, with *Curcuma longa *(turmeric); Group D, with kumkum; Group E, with *Hibiscus rosa-sinensis *(hibiscus); Group F, with *Beta vulgaris *(beetroot); and Group G, with eosin as a control. Each staining group comprised tissue sections from four distinct oral histopathologies: normal tissues, oral squamous cell carcinoma, ameloblastoma, and odontogenic cysts. Five cases were selected for each histopathological type, and each tissue sample was sectioned into seven slides for staining with seven different stains, resulting in a total of 140 slides (five tissue sections × seven stains) (Figures 1-4).

The interobserver reliability was assessed using intraclass correlation coefficient (ICC) analysis, which yielded a value of 0.96, indicating excellent agreement between the two observers.

The study aimed to compare the effects of different stains on tissue samples by analyzing seven groups (A-G). Statistical analysis was conducted using ANOVA, followed by post-hoc Tukey's honestly significant difference (HSD) analysis, to determine significant differences in mean values between the groups.

Among the tissue sections stained using seven different dyes, a one-way ANOVA test revealed a highly statistically significant difference among the groups (p=0.001) (Table 2). Group G, representing eosin-stained sections (control; m=15.85), demonstrated the highest mean value, indicating its superior staining performance. Sections stained with *Lawsonia inermis *(m=15.05), though slightly lower than the control, showed comparable results without a significant difference (p>0.05). Group D, stained with kumkum (m=13.5), demonstrated intermediate staining intensity, showing a statistically significant difference compared to the control (p<0.05) but with better results than other natural dyes. Group B, stained with *Zingiber officinale *(m=9), showed significantly lower staining intensity compared to eosin (p<0.001). Group C, stained with *Curcuma longa *(m=12.3), provided moderate staining, but the results were significantly inferior to the control (p<0.05). Group E, stained with *Hibiscus rosa-sinensis* (m=7.9), and Group F, stained with *Beta vulgaris *(m=8.4), exhibited the lowest mean values, significantly differing from eosin.

**Table 2:** One-way ANOVA test between the natural stains. P<0.01 is significant Group A: *Lawsonia inermis *(henna). Group B: *Zingiber officinale *(ginger). Group C: *Curcuma longa *(turmeric). Group D: kumkum. Group E: *Hibiscus rosa-sinensis *(hibiscus). Group F: *Beta vulgaris *(beetroot). Group G: eosin as a control

Group	N	Mean	SD	F-value	P-value
Group A	4	15.05	0.3416	27.74	0.001
Group B	4	9	1.4236		
Group C	4	12.3	2.2061		
Group D	4	13.5	1.8788		
Group E	4	7.9	0.7746		
Group F	4	8.4	0.1633		
Group G	4	15.85	0.1		

Post-hoc Tukey's HSD analysis was performed to assess pairwise comparisons among the seven staining groups. The results revealed that eosin (Group G) exhibited the highest and most consistent staining efficacy, significantly outperforming most natural dyes. Among the natural dyes, *Lawsonia inermis *(Group A) was the only one comparable to eosin, showing no significant difference in staining performance, indicating its equivalence to eosin. Kumkum(Group D) demonstrated moderate staining performance, comparable to *Lawsonia inermis *and approaching the efficacy of eosin, while significantly outperforming the other natural dyes. *Zingiber officinale *(Group B), *Curcuma longa *(Group C), *Hibiscus rosa-sinensis *(Group E), and *Beta vulgaris *(Group F) exhibited significantly lower staining efficacy compared to eosin, with hibiscus and beetroot performing the poorest. Post-hoc Tukey's HSD comparative analysis between Group G (eosin) and other stains is depicted in Table 3. In summary, Group G (control) appears to perform as expected, maintaining a high mean value and low variability. Group A closely matches the control and may serve as an alternative in certain scenarios. Groups B, C, E, and F differ significantly from the control, suggesting these stains may not provide equivalent results to eosin.

**Table 3:** Post-hoc Tukey's HSD analysis between Group G (eosin) and other groups. P<0.01 is significant Group A: *Lawsonia inermis *(henna). Group B: *Zingiber officinale *(ginger). Group C: *Curcuma longa *(turmeric). Group D: kumkum. Group E: *Hibiscus rosa-sinensis *(hibiscus). Group F: *Beta vulgaris *(beetroot) HSD: honestly significant difference

Comparison	Mean difference	P-value
Group G vs Group A	-0.9578	0.4307
Group G vs Group B	7.0966	0.0000
Group G vs Group C	3.4970	0.0000
Group G vs Group D	1.8103	0.0146
Group G vs Group E	8.5460	0.0000
Group G vs Group F	7.4706	0.0000

## Discussion

Staining is a vital part of histology; however, the use of synthetic stains poses potential threats to the ecosystem. Therefore, we explored naturally available stains as potential substitutes for eosin. In the present study, six natural dyes, including *Zingiber officinale* (ginger), *Lawsonia inermis *(henna), *Curcuma longa *(turmeric), *Beta vulgaris *(beetroot), kumkum, and *Hibiscus rosa-sinensis *(hibiscus), were assessed in comparison with eosin in various oral histopathologies. The objective of this study was to evaluate the diagnostic efficacy of natural dyes in identifying oral histopathologies compared to eosin.

Among the natural stains examined in this study, *Lawsonia inermis *(henna) demonstrated superior results, with staining efficiency nearly equivalent to that of eosin in diagnosing the pathologies. *Lawsonia *provided excellent contrast and good morphological detail of the cytoplasm and nucleus, with other structures like collagen, salivary glands, and bone clearly visible in the tissue sections. Raju et al. evaluated *Lawsonia inermis *as a substitute for eosin in staining normal oral mucosa and oral squamous cell carcinoma tissue, finding it 15% less efficient than eosin [6]. Meenakshi et al. compared turmeric, hibiscus, and henna stains with eosin in oral cytology, concluding that henna offered excellent nuclear detail, good cytoplasmic detail, and clarity, but its staining intensity was inferior to eosin [7]. Henna leaves (*Lawsonia inermis*) contain the red-brown dye lawsone (2-hydroxy-1,4-naphthoquinone, C₁₀H₆O₃), which is present at concentrations ranging from 0.5% to 2%, contributing to its pigmentation properties [8]. In the present study, henna served as a cytoplasmic stain, revealing excellent nuclear detail, clear cytoplasmic features, and overall clarity in both normal mucosa and pathological tissue sections.

Kumkum, a natural dye, showed moderate results in the tissue sections of the present study. It provided excellent cytoplasmic and nuclear details and stained acellular structures well, though with moderate contrast in tissue sections. Navya et al. stated that kumkum has better staining characteristics and overall performance than turmeric and eosin in histopathology sections of cervix tissue, concluding that kumkum is an efficient counterstain for various structures [5]. Thajudeen et al. evaluated kumkum as a substitute for eosin in staining tissue sections from the normal cervix, endometrium, ovaries, arteries, smooth muscle, and nerve bundles. They concluded that kumkum is an effective counterstain, enhancing the visualization of red blood cells (RBCs) in arteries and cell cytoplasm in the glandular and squamous epithelium as well as, or better than, eosin [9]. Lavanya et al., in their study on kumkum made from *Curcuma aromatica *extract and slaked lime, found it to be a noteworthy alternative to traditional H&E staining. They highlighted its similar staining characteristics, cost-effectiveness, eco-friendliness, non-allergenic and non-carcinogenic nature, and easy availability [10]. Kumkum, derived from the saffron flowers of *Crocus sativus* L., contains 150 volatile compounds and numerous non-volatile compounds. Key components include crocin, which imparts color; picrocrocin, responsible for bitterness; and safranal, which provides a distinctive aroma. Commercially available kumkum powder varies by brand but typically includes turmeric powder, chalk powder, calcium salts, and saffron [11].

In the present study, other natural dyes such as *Curcuma longa *(turmeric) produced moderately satisfactory results. While turmeric offered optimal contrast at low magnification, the cytoplasmic and nuclear details were somewhat obscured by the counterstain. *Zingiber officinale *(ginger) exhibited low staining intensity, providing moderate contrast and nuclear detail but less effective cytoplasmic resolution. *Beta vulgaris *(beetroot) yielded sub-optimal mean values in terms of staining quality. Among all the natural stains used, *Hibiscus rosa-sinensis *demonstrated the weakest performance, showing inferior results when compared to other natural dyes and eosin. Both *Beta vulgaris *and *Hibiscus rosa-sinensis *exhibited low staining intensity, with hibiscus providing poorer contrast and less detailed cytoplasmic and nuclear resolution than beetroot.

Contrary to the present study, Sudhakaran et al. found that ginger and turmeric extracts demonstrated staining potential similar to eosin, with *Zingiber officinale *outperforming *Curcuma longa *and closely resembling eosin [3]. Although *Zingiber officinale *and *Curcuma longa *in the present study showed moderately good results, their staining intensity did not match that of eosin and *Lawsonia inermis*. The contrasting results in the present study might be due to variations in extraction techniques and differences in staining protocols such as pH, incubation time, etc. Ajileye et al. observed that *Zingiber officinale *ethanolic extract, used as a counterstain with alum hematoxylin, effectively stained muscle fibers and cytoplasm yellow in Wistar rat tissues, showing promise as a natural dye [12]. Sridhara et al. concluded that hibiscus extract is comparable to eosin for staining oral mucosal and skin tissues, contrasting with the present study, likely due to differences in extract preparation and staining methods [13]. Various studies have utilized beetroot extract as a histological stain for diagnosing organisms like intestinal nematode ova [3], fungal samples (*Rhizopus *sp., *Microsporum gypseum*, *Aspergillus niger*), and buccal smears [14,15]. These studies reported poor staining efficacy for nematodes, satisfactory clarity for fungi, and good cellular morphology in buccal smears. However, the present study found beetroot alcoholic extract provided the least contrast and very poor cellular and nuclear details compared to *Lawsonia inermis *and eosin.

To the best of our knowledge, this is the first study to evaluate and compare the staining effectiveness of six natural dyes with eosin across various oral histopathological tissue sections. This investigation provides a comprehensive assessment of the potential of natural dyes as alternatives to eosin in histopathological staining. Among the natural stains evaluated, *Lawsonia inermis *(henna) emerged as a standout performer, exhibiting staining efficiency comparable to eosin across normal oral mucosa and pathological tissue sections, with excellent contrast and detailed morphological features. Kumkum, another natural dye examined in this study, demonstrated promising results, showcasing good staining efficiency and enhancing the visualization of various tissue structures. While its staining intensity did not match that of *Lawsonia inermis *or eosin, kumkum exhibited sufficient contrast and definition, making it a viable candidate for further investigation. Its ability to highlight cellular details suggests its potential as a complementary stain in histopathological applications. Turmeric, ginger, *Beta vulgaris *(beetroot), and *Hibiscus rosa-sinensis*, while explored as potential alternatives, presented varying degrees of staining efficacy, with some falling short in contrast and detailed cellular resolution compared to *Lawsonia inermis *and eosin. Their optimization can be done through modifications in extraction, pH adjustments, and mordant applications which could enhance their performance and broaden their use in histopathology.

The limitations of the present study include the lack of evaluation of the long-term stability and shelf life of the extracted natural stain, which may impact its practical applicability in routine diagnostics. Additionally, variations in environmental factors, such as temperature and humidity during dye extraction and storage, could influence the reproducibility of the results. Beyond these limitations, future research should aim to optimize extraction and storage techniques to improve the stability and shelf life of natural dyes, ensuring their reliability for routine histopathological applications. Standardizing staining protocols, such as pH regulation and mordant usage, may enhance reproducibility and staining efficiency. Expanding studies to include a larger sample size and a wider range of tissue types would offer a more thorough assessment of these dyes' diagnostic potential. Moreover, investigating the biochemical interactions between natural dyes and tissue components could further refine their application and support their adoption as viable alternatives to synthetic stains.

## Conclusions

These findings of the present study underscore the potential of *Lawsonia inermis *as a viable substitute for eosin in histopathological staining protocols, offering comparable staining efficacy and potentially widening the spectrum of available staining options in diagnostic pathology. Further research and validation are warranted to explore the clinical utility and broader applicability of these natural dyes in routine histopathological practice. Additionally, discrepancies observed in staining efficacy among natural dyes in different studies highlight the importance of standardized protocols and rigorous evaluation to ensure reproducibility and reliability in histopathological analyses.
